# Differential contribution of working memory to auditory rhythm discrimination in stuttering and nonstuttering adults

**DOI:** 10.21203/rs.3.rs-6686913/v1

**Published:** 2025-06-11

**Authors:** Emily Garnett, Toni Smith, Bailey Rann, Nicholas Mularoni, Soo-Eun Chang, J. Devin McAuley

**Affiliations:** University of Michigan-Ann Arbor; Michigan State University; Michigan State University; University of Michigan-Ann Arbor; University of Michigan-Ann Arbor; Michigan State University

**Keywords:** Fluency, Rhythm, Timing, Speech, Basal Ganglia

## Abstract

Stuttering is a neurodevelopmental condition characterized by involuntary disruptions in the rhythmic flow of speech. Notably, stuttering is associated with aberrant structure and function of the basal ganglia thalamocortical network. Separately, the BGTC network has been implicated in non-speech beat and rhythm perception. Supporting a link between the two sets of findings, children who stutter exhibit poorer auditory rhythm discrimination compared to non-stutterers, especially for complex rhythms without a consistently marked beat. For adults who stutter (AWS), data showing a link between stuttering and poorer auditory rhythm discrimination has been mixed. One possible reason may be that AWS have developed strategies for rhythm discrimination that leverage an alternative non-BGTC network dependent timing mechanism. One candidate from the timing literature is the use of an interval-based mechanism that involves the cerebellum. From this perspective, rhythm discrimination judgments for AWS would involve interval-by-interval duration comparisons, which should be expected to place a greater burden on working memory compared to the more automatic beat-based timing processes implemented by the BGTC network. To investigate this hypothesis, we combined data from three studies where AWS and age-matched controls performed the same rhythm discrimination and working memory tasks. Across studies, AWS, as hypothesized, showed a significantly stronger positive correlation between working memory and rhythm discrimination than controls where there were no (or very weak) correlations. Moreover, separate group comparison of rhythm discrimination performance for AWS with high and low working memory scores reveals no difference between controls and AWS with high working memory scores, but much poorer performance by AWS with low working memory scores compared to controls. These results support the view that AWS may mask difficulties in rhythm perception and an underlying impairment in beat-based timing by leveraging a distinct working memory dependent interval timing mechanism to discriminate rhythms.

## Introduction

Developmental stuttering is a neurodevelopmental condition that affects speech motor control resulting in characteristic disruptions in the temporal flow of speech such as sound/syllable repetitions, sound prolongations, and silent blocks. The onset of developmental stuttering typically occurs between two and five years of age^1,2^ during a period of rapid growth in speech, language, cognition, and social and emotional skills^3^. Stuttering develops in up to 8 percent of children, becoming a lifelong chronic condition for approximately 1 in 5 adults who stuttered as a child^2^.

Over two decades of neuroimaging findings support the view that the neurobiological bases for stuttering is rooted in aberrant structure and function of the basal ganglia thalamocortical network ([BGTC]; for reviews, see^4,5^). Key findings from this work include atypical patterns of connectivity in individuals who stutter within corticostriatal circuits as shown by structural and functional MRI studies ^6–10^, evidence of impaired basal ganglia function in adults who stutter compared to controls^8,11^, and structural differences in cortical regions involved in speech production such as the left inferior frontal and premotor cortices^6,9,12–16^. Children who would go on to have persistent stuttering showed significant gray matter volume decreases relative to peers who don’t stutter in the bilateral ventral striatum including the putamen^15^. A longitudinal study by Chow & Chang^17^ further found that stuttering severity in children correlates with white matter integrity along superior and anterior thalamic radiations. Stuttering persistence was associated with slower growth rate of white matter tracts connecting frontotemporal, motor, and basal ganglia regions. Children with persistent stuttering also showed significantly reduced activity during speech preparation in the putamen compared to children who do not stutter, and this difference increased with age^14^. Collectively, these studies contribute to the understanding that stuttering is associated with disruptions in the basal ganglia thalamocortical (BGTC) network, influencing motor control areas responsible for fluent speech production.

The BGTC network has also been implicated in non-speech rhythm perception and production through its implementation of a beat-based timing mechanism whereby putative neural oscillators are entrained (synchronized) by the periodic beat of rhythmic stimuli, enabling the metric encoding of rhythm and efficient predictions/judgments about stimulus timing. From this perspective, the supplementary motor area (SMA) along with the putamen have been proposed to form a medial timing circuit that supports beat-based timing^18–22^. Empirical evidence substantiating involvement of the medial timing circuit in beat-based timing comes from neuroimaging studies which show greater activation of these areas during the presentation of rhythms that have a strongly implied beat compared to rhythms that have a weak or absent beat^18,20,23^. Furthermore, individual differences in beat perception have been shown to be correlated with SMA activation^19^ and basal ganglia impairment in Parkinson Disease is associated with deficits in beat-based rhythm discrimination^24^.

Taken together, research showing (1) aberrant functioning of the BGTC network in developmental stuttering and (2) involvement of the BGTC network in rhythm perception has led to the hypothesis that individuals who stutter have a deficit in the ability to internally generate a beat, disrupting the temporal control of speech production^25–27^. This *internal beat deficit hypothesis* for stuttering posits that individuals who stutter have difficulties with the internal timing mechanisms needed to produce fluent speech. From this perspective, stuttering may arise from a disruption in the ability to generate internal rhythmic patterns, or “beats,” which are crucial for the encoding of hierarchical (metrical) structure and the timing and coordination of speech-related motor activities. In typical speech production, these nested internal rhythms help coordinate the intricate timing needed to initiate and maintain smooth speech flow. The internal beat deficit hypothesis implies that people who stutter may have an impaired ability to use these timing cues effectively, leading to disruptions in speech fluency.

Support for an *internal beat deficit* in developmental stuttering comes from several sources. Individuals who stutter become temporarily fluent in conditions where there is an external timing referent that can substitute for an internal periodic beat, for example while synchronizing speech to a metronome, speaking in unison with another person (“choral speech”), or while singing^28–32^. Evidence for differences in rhythm and timing abilities between children who stutter (CWS) and non-stuttering children has been relatively consistent: CWS show more variable and/or poorer performance on speech and non-speech rhythm production and perception tasks such as tapping, clapping, and rhythm discrimination relative to their fluent peers^26,33–36^. Wieland et al.^26^ examined rhythm perception in CWS and non-stuttering children and found that CWS demonstrate worse auditory rhythm discrimination than their non-stuttering peers for complex rhythms that do not have a consistently marked beat. Chang et al.^33^ extended this work by showing that the strength of resting-state functional connectivity in the BGTC network predicts rhythm discrimination performance for typically developing children but not for children who stutter.

Research on rhythm perception and production in adults who stutter (AWS) has been much less conclusive. In favor of the presence of an internal beat deficit in AWS, some studies have found that AWS exhibit greater temporal variability in self-paced speech production and finger tapping^37^, less precise coupling of effectors in a rhythmic bimanual coordination task^38^, and poorer performance in discriminating complex rhythms that do not have a consistently marked beat for more severe stutterers compared to less severe stutterers^27^. Other studies have found the opposite pattern, such as overall slower and *less* variable self-paced motor production in AWS compared to controls.^39^ Still other studies have shown no group differences. AWS have been found to not differ from controls in accuracy or variability when synchronizing with or continuing the pace of a metronome, whether doing so with speech, non-speech facial movements, or a complex finger movement, and at a variety of rates^40^. Similar null results were found for synchronized finger tapping and side-to-side finger movements^41,42^. Further supporting no group difference in the speech timing domain, Assaneo et al.^43^ found that AWS and controls showed a similar ability to synchronize produced speech to heard speech.

One reason for the inconsistent findings in adults may be that over the course of their lives, AWS have developed compensatory strategies that enable them to at least partially overcome inherent timing limitations associated with a compromised BGTC network, such as leveraging an alternative timing mechanism that does not require the generation of an internal beat. One possible candidate from the literature is the use of an interval-based timing mechanism that involves the cerebellum and is associated with the representation of absolute durations^44–46^. From an interval-timing perspective, successive time intervals comprising a rhythm are measured (timed) independently and rhythm-discrimination judgments are based on interval-by-interval comparisons of absolute durations. In the literature, the brain regions associated with interval-based timing appear to be distinct from those supporting beat-based timing^18–20,47–50^. Indeed, there is evidence for a double dissociation between the basal ganglia and cerebellum in terms of the disruption of timing functions: cerebellar degeneration is associated with deficits in the absolute timing of single time intervals but beat-based timing is unaffected, while basal ganglia impairment in Parkinson’s disease is associated with deficits in beat-based timing but unaffected single-interval timing^24,48,50^. Even in neurologically unaffected individuals, the cerebellum and BGTC network are differentially activated in tasks that target interval or beat-based timing, respectively^49^.

There is some converging support that AWS may compensate for a deficit in the BGTC network and beat-based timing by leveraging the cerebellum and an associated interval-based timing mechanism. Recent work by Garnett et al.^27^ found that AWS exhibited increased activity in beat-based timing areas (basal ganglia, SMA) relative to controls during rhythm discrimination, particularly for complex rhythms that lack a consistently marked beat, despite somewhat lower task performance for complex rhythms in AWS compared to controls. This enhanced activity may reflect greater but less efficient recruitment of these areas in AWS. Additionally, enhanced functional connectivity observed between the basal ganglia and cerebellum for simple relative to complex rhythms in AWS suggests greater recruitment of an interval-based timing mechanism. Additional support for the potential use of an alternative interval-based timing mechanism to discriminate rhythms by AWS comes from several sources. Research suggests that the cerebellum may also become hyperactive as a compensatory mechanism for deficits in other brain regions involved in speech production^11,51–57^. A recent study found that AWS exhibit increased functional connectivity within the cerebellum when pacing their speech with an external metronome (vs. during internally paced speech)^53^. Connectivity changes (primarily reductions) have also been associated with treatment or recovery from stuttering^55,57–60^.

The cerebellum, traditionally known for its role in timing, motor coordination, and balance, has also been implicated in cognitive functions, including working memory^61,62^. The cerebellum has extensive connections with the prefrontal cortex, a region critical for working memory. Through these connections, the cerebellum may influence cognitive processes by modulating activity in the prefrontal cortex, especially in tasks requiring the maintenance and manipulation of temporal information^63^. Consequently, viewing rhythm discrimination from an interval-timing perspective suggests that rhythm discrimination would involve interval-by-interval comparison of the rhythms, a process that would tax working memory. Models that adopt an interval-based approach to timing typically hypothesize an internal pacemaker-like mechanism that continuously emits pulses that are ‘counted’ by an attention-mediated accumulator. Interval durations are then stored in memory as the accumulated count or duration code, with greater pulse counts corresponding to longer perceived intervals ^64–67^. Discrimination of single interval durations then requires maintaining in working memory the duration code for a standard interval in order to compare it to another interval. Similarly, rhythm discrimination would involve maintaining the set of duration codes corresponding to the sequence of intervals that comprise the standard rhythm in working memory, and then checking each one against the corresponding interval in the comparison rhythm.

One implication of this view is that if AWS rely on the use of an interval timing mechanism and interval-by-interval duration comparisons to make discrimination judgments, we would expect a stronger relationship between working memory and rhythm discrimination performance in AWS compared to non-stuttering adults. This is because the use of interval timing to discriminate rhythms would require maintaining each interval comprising a standard rhythm in working memory and making interval-by-interval duration comparisons. Conversely the use of a beat-based timing mechanism to discriminate rhythms affords the metric encoding of rhythm and efficient predictions/judgments about stimulus timing based on the detection of phase differences between entrained beats and stimulus events^68^. To investigate this hypothesis, we combined data from three different studies of rhythm processing in AWS that each obtained the same measures of working memory and auditory rhythm discrimination. We then considered the relation between working memory capacity and rhythm discrimination performance separately for AWS and control participants with the prediction that AWS would show a greater positive correlation between working memory and rhythm discrimination performance than the control group.

## Methods

### Participants

The combined participant sample from the three studies consisted of 159 participants: 94 non-stuttering adults (hereafter: controls), ages 18–60 years (*M* = 27.4, *SD* = 9.5; n = 42, female) and 65 AWS, ages 18–55 years (*M* = 28.2, *SD* = 9.6; n = 16, female). Common to all three studies, participants spoke American English as their primary language and reported no speech, language, cognitive, or hearing conditions other than stuttering in the AWS group. In addition, controls reported no personal or family history of stuttering. Stuttering severity for AWS was assessed according to the Stuttering Severity Instrument 4th edition ([SSI-4]^69^) by a certified speech-language pathologist with expertise in stuttering. The mean SSI score was 24.52 (*SD* = 9.04) and ranged from 9 (very mild) to 45 (very severe). All participants provided informed consent and the research was performed in accordance with relevant guidelines/regulations and the Declaration of Helsinki. Participants received compensation for their time. Below we describe participant details for each of the three studies separately; see Table 1 for additional participant details and group comparisons.

Study 1 consisted of data from Garnett et al. (2023)^27^, which comprised 34 participants: 17 controls, ages 19–44 years (*M* = 25.1, *SD* =7.1; n = 6, female) and 17 AWS, ages 18–53 years (*M* = 28.2, *SD* = 10.9; n = 5, female). Participants were right-handed, reported no developmental, neurological, or psychiatric conditions, and were not taking any medication affecting the central nervous system. Groups did not differ in years of education or self-reported years of formal musical training. Mean SSI-4 score for the AWS group was 26.24 (*SD* = 7.45, range 14–37). All research procedures were approved by the Michigan State University Institutional Review Board.

Study 2 consisted of data from 53 participants recruited for a randomized controlled trial ([RCT]; NCT03437512). There were 26 control participants ages 18–56 years (*M* = 25.4, *SD* =9.7; n = 18, female) and 27 AWS ages 19–49 years (*M* = 25.5, *SD* = 7.3; n = 3, female). Groups did not differ in years of education or musical training as measured using the Goldsmiths Musical Sophistication Index ([GMSI]^70^). Mean SSI-4 score for the AWS group was 24.44 *(SD* = 8.34, range 12–40). Participants reported no developmental, neurological, or psychiatric conditions, and were not taking any medication affecting the central nervous system. All research procedures were approved by the Institutional Review Boards of the University of Michigan Medical School.

Study 3 consisted of data from 72 participants from an ongoing RCT (NCT04929184) investigating rhythm processing in AWS. There were 51 control participants ages 18–60 years (*M* = 29.1, *SD *= 10.0; n = 18, female) and 21 AWS ages 18–55 years (*M* = 31.6, *SD* = 10.3; n = 8, female). Mean SSI-4 score for the AWS group was 23.24 (*SD* = 11.08, range 9–45). Groups did not differ in years of education or musical training as measured using the GMSI. Participants reported no developmental or neurological conditions. Participants with self-reported anxiety or depression (with or without medication) were included in this study, with similar numbers in each group. All research procedures were approved by the Institutional Review Boards of the University of Michigan Medical School and Michigan State University.

## Experimental Tasks

### Working Memory

Working memory capacity was measured using the automated complex Operation Span (OSPAN) Task^71^ presented with E-Prime v2 (Psychology Software Tools, Inc.), except for in Study 3 where E-Prime v3 was used. Participants across the datasets completed the same version of the OSPAN task, during which they solved a series of simple arithmetic equations while remembering lists of unrelated letters.

#### Rhythm Discrimination

##### Task.

Participants completed the same auditory rhythm discrimination task in all three studies. On each trial, participants heard two successive presentations of a standard rhythm and judged whether a third comparison rhythm was the same or different from the standard by pressing one of two buttons labeled ‘same’ or ‘different.’

##### Stimuli.

Rhythms for all three studies were a subset of those used in Grahn and Brett^24^. Rhythms were 5, 6, or 7 intervals long and all intervals within a rhythm were integer multiples of a base time unit, which varied randomly from trial to trial, taking on values of 220, 245, or 270 ms. The frequency of the tones marking the rhythms was fixed within a trial, but also varied randomly from trial to trial, taking on one of six values: 294, 353, 411,470, 528, or 587 Hz. There were two rhythm types: simple and complex. Simple rhythms were composed of intervals organized in such a way that a tone always occurred every four base time units, consistently reinforcing an explicit beat. In contrast, complex rhythms were composed of intervals organized in such a way that a tone did *not* always occur every four base time units, resulting in sequences where the temporal location of the tones did not consistently reinforce the explicit beat. Based on prior studies, the simple rhythms were expected to induce a stronger perception of a periodic beat in listeners (and better rhythm discrimination performance) than complex rhythms^72^. In order to control the interval composition of the sequences across rhythm type, each simple rhythm had a matched complex rhythm that consisted of the same set of interval durations, but presented in a different order. The ‘different’ variant of each rhythm involved a modification of the corresponding standard rhythm, which swapped the order of a pair of adjacent intervals. The different variants used here were the same as those used in Grahn and Brett^24^.

Study 1 consisted of 12 simple and 12 complex auditory rhythms. Study 2 consisted of 15 simple and 15 complex auditory rhythms. Study 3 consisted of a different subset of 15 simple and 15 complex auditory rhythms than Study 2. Additionally, the stimuli used in Study 3 consisted of “filled” intervals (i.e., the intervals were marked by long tones with brief silences in between) while the stimuli used in Study 1 and Study 2 consisted of “unfilled” intervals (i.e. the intervals were marked by brief 50-ms tones with longer silences in between).

##### Procedure.

Participants completed the rhythm discrimination task for Study 1 while undergoing fMRI using E-Prime v2.0 Professional (Psychology Software Tools, Inc.) to control stimulus presentation and response collection. Trials were in six blocks lasting approximately seven minutes each. Each block consisted of 24 trials in which participants heard both same and different variants of the 12 simple and 12 complex rhythms. The order of stimuli was determined by creating three sets of randomized orders of all 12 simple and 12 complex rhythms. In each set, half of the trials were ‘same’ trials (the correct response was same) and half of the trials were ‘different’ trials (the correct response was different). Participants had 2100 ms to respond using an MR-compatible keypad before the next trial started.

For Study 2 and Study 3, participants completed the rhythm discrimination task in a quiet room in front of a laptop or desktop computer with E-Prime v2.0 (for Study 2) or v3.0 (for Study 3) Professional (Psychology Software Tools, Inc.) controlling stimulus presentation and response collection. The trials were in two blocks lasting approximately 15 minutes each with each block consisting of 30 trials. Across the two blocks, participants heard both same and different variants of each of the 15 simple and 15 complex rhythms. The order of the stimuli was randomized across trials, with half of the trials consisting of ‘same’ trials (the correct response was ‘same’) and half of the trials consisting of ‘different’ trials (the correct response was ‘different’). Trials were self-paced, and participants could not move on to the next trial until they provided a response. Responses were selected by pressing labeled keys on a computer keyboard, with 1 for “same” and 2 for “different.”

#### Data Analyses

##### Working Memory.

The primary dependent measure used for the OSPAN task was the number of letters remembered in the correct order calculated for each participant (referred to as the Partial score). Potential study and group differences in working memory were analyzed using a 3 (Study: 1, 2, 3) x 2 (Group: AWS vs Controls) between-subjects ANOVA.

##### Rhythm Discrimination.

Rhythm discrimination performance was assessed by analyzing ‘different’ response proportions for trials where the comparison rhythm was different from the standard rhythm (a hit) and for trials where the comparison rhythm was the same as the standard rhythm (a false alarm) and then using the resulting hit and false alarm rates to calculate the signal detection measure of sensitivity, *d’* ^73^. Potential differences in rhythm discrimination performance across conditions were evaluated using a 3 (Study: 1,2, 3) x 2 (Group: AWS vs Controls) x 2 (Rhythm Type: Simple vs Complex) mixed-measures ANOVA on *d’* Additionally, because we had specific a priori hypotheses regarding performance on this task, we also conducted planned between-group comparisons for simple and complex rhythms.

##### Relation between Working Memory and Rhythm Discrimination.

To assess the relationship between working memory capacity and rhythm discrimination performance in the combined dataset, Pearson correlations were calculated between the OSPAN scores and simple and complex rhythm discrimination performance. A Fischer r-to-z transformation was used to test the significance of any differences in correlations between groups. Because we had a clear, hypothesis driven prediction (i.e., AWS would demonstrate stronger correlations compared to controls), one-tailed tests were used.

##### Treatment of Duplicate Study Enrollment

Three individuals participated in more than one of the three studies. For the analyses of the combined data set reported below, each participant was only included *once* to eliminate duplicate data. Separate analyses for each study reported in the Supplementary Material included the three individuals with duplicate enrollment across studies.

## Results

### Working Memory

A 3 (Study) x 2 (Group) ANOVA on OSPAN scores revealed a main effect of Study, *F*(2, 150) = 20.68, *p*< 0.001, *η*^*2*^ = 0.22, but no main effect of Group, *F(1,* 150) = 1.51, *p* = 0.221, *η*^*2*^ =0.01, and no interaction between Study and Group, *F*(2, 150) = 0.61, *p*= 0.545, *η^2^* = 0.01. Overall, OSPAN scores did not significantly differ between AWS and control participants (AWS, *M* = 51.81, *SD *= 15.65; Control, *M* = 55.24, *SD* =15.62). Post-hoc analyses of the main effect of Study revealed overall lower OSPAN scores in Study 1 (*M* = 39.63, *SD* = 17.26) compared to Study 2 (*M* = 56.69, *SD* = 12.73), *t*(82) = 5.20, *p* <0.001, Cohen’s *d* =1.17) and compared to Study 3 (*M*= 58.13, *SD* =13.19), *t*(102) = 5.98, *p*< 0.001, Cohen’s *d* = 1.27).

#### Rhythm Discrimination

**Figure 1:**
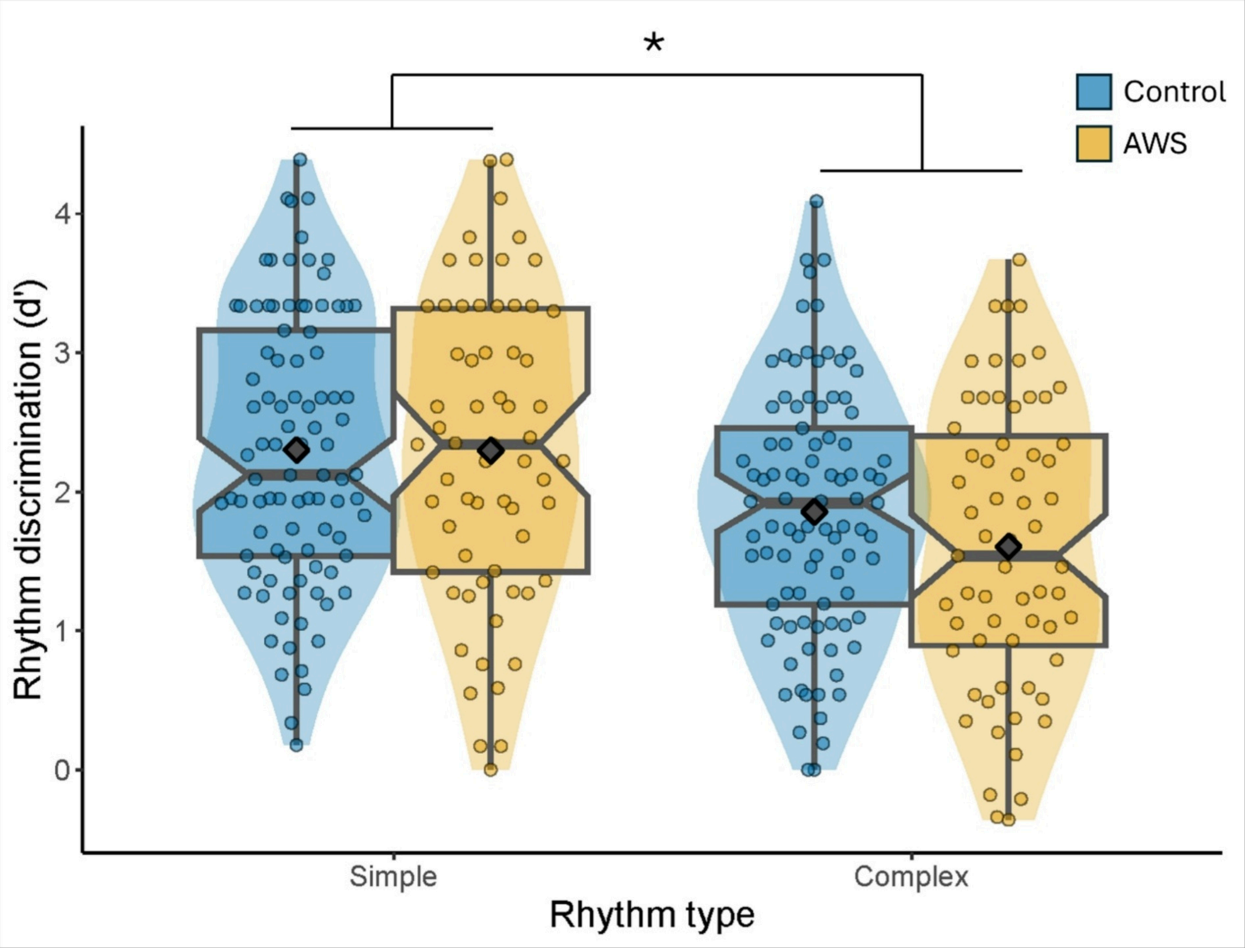
Comparison of Rhythm Discrimination Across Conditions and Groups. Violin plot illustrating the distribution and density of rhythm discrimination scores (d-prime) across two rhythm types: “Simple” and “Complex”. The data is further stratified by Group, with “Control” and “AWS” participants represented in blue and yellow, respectively. Overlaid boxplots provide medians and quartile ranges. Superimposed gray diamonds indicate mean scores for each condition and group combination. Simple rhythms were discriminated significantly better than complex rhythms, as indicated by the asterisk.

A 3 (Study) x 2 (Group) x 2 (Rhythm Type) ANOVA on rhythm discrimination scores (*d*’) revealed no main effects of Study, *F*(2,150) = 0.82, *p* = 0.442, *η*^*2*^ = 0.01, or Group, *F*(1,150) = 0.74, *p* = 0.390, *η* = 0.005, but the expected main effect of Rhythm Type, *F*(1,150) = 140.68 *p* <0.001, *η^2^* = 0.48, and a significant interaction between Rhythm Type and Study, *F*(2,150) = 19.23, *p*< 0.001, *η^2^* = 0.20. The interaction between Rhythm and Group did not reach statistical significance but showed the expected advantage of simple over complex rhythm discrimination. Additionally, there was trend towards poorer performance by AWS compared to control participants, especially for complex rhythms, *F*(1,150) = 3.62, *p* = 0.059, *η^2^* = 0.024. Overall, simple rhythms were better discriminated than complex rhythms for both groups (simple: *M* = 2.30, *SD*=1.02; complex: *M* = 1.75, *SD*= 0.96; Fig. 1). AWS also showed slightly worse complex rhythm discrimination compared to controls (AWS: *M* = 1.61, *SD*=1.03; Controls: *M*= 1.85, *SD*= 0.90), but not significantly so, t(154) = 1.60, *p* =0.113, Cohen’s *d* = 0.26. Simple rhythm discrimination did not differ between groups (AWS: *M* = 2.35, *SD* =1.09; Controls: *M* = 2.38 *SD* = 0.97), t(154) = 0.03, *p* = 0.975, Cohen’s *d*= 0.01.

### Relation between Working Memory and Rhythm Discrimination

**Figure 2:**
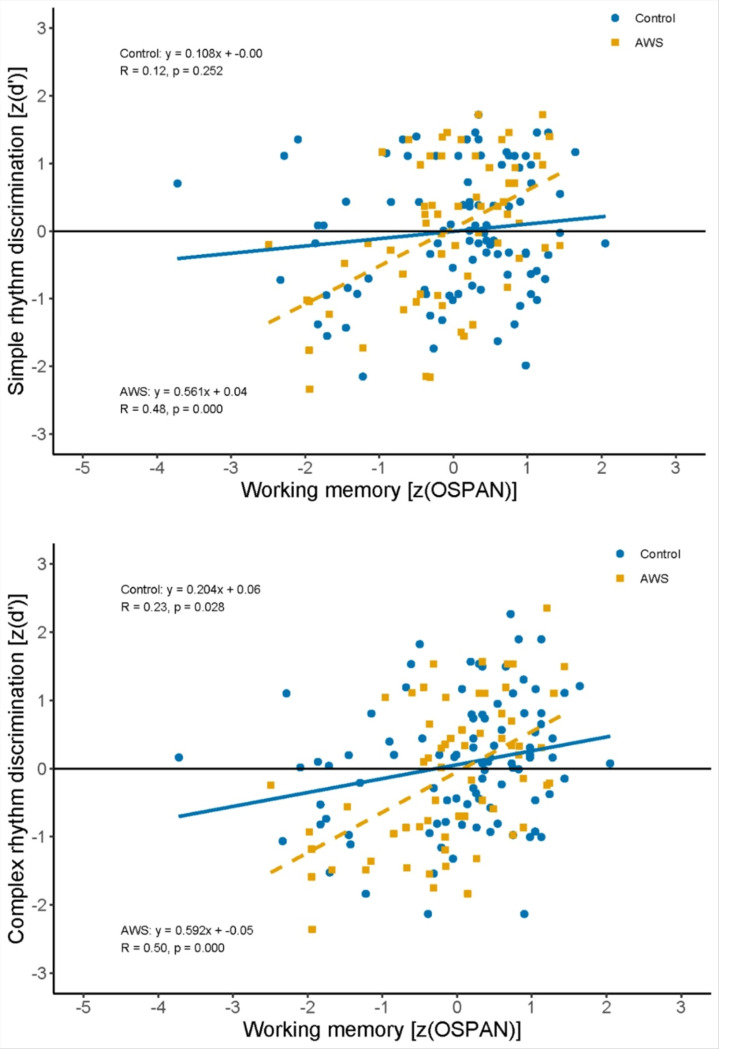
Relation between Working Memory and Rhythm Discrimination. Scatter plots showing the relation between the OSPAN measure of working memory and simple rhythm discrimination (top) and complex rhythm discrimination bottom for both AWS (yellow squares) and Controls (blue circles). Z transformed OSPAN scores are on the x-axis and Z transformed d’ scores are on the y-axis.

Next, to test the main hypothesis, we examined the relationship between working memory and rhythm discrimination separately for AWS and controls. To combine the data across studies, OSPAN and rhythm discrimination scores were first z-transformed within each study in order to control for overall performance differences between studies. Pearson correlations were then calculated between transformed OSPAN and rhythm discrimination scores for both participant groups for simple and complex rhythm discrimination separately. The upper panel of Fig. 2 shows that simple rhythm discrimination was positively correlated with working memory for AWS, *r*(61) = 0.475, *p*< 0.001, but not for controls, *r*(91) = 0.120, *p* = 0.252. The lower panel of Fig. 2 shows that complex rhythm discrimination was positively correlated with working memory for both AWS (*r*(63) = 0.501, *p*< 0.001) and controls (*r*(91) = 0.228, *p* = 0.028). In both cases, there was a stronger positive correlation between working memory and rhythm discrimination for AWS compared to controls. A Fisher r-to-z transformation showed that the difference in correlation between groups was significant for both simple rhythms (*z* = 2.38, *p* = 0.0044) and complex rhythms *(z*= 1.91, *p* = 0.0281, one-tailed). Separate analyses of the data from each of the three studies yielded a similar pattern of results (see Supplementary Material). One control participant from Study 3 had an OSPAN score that was more than 3 SD below the Study 3 mean. To consider whether this participants’ exceptionally low OSPAN score may have influenced the results, we re-calculated the correlations and Fischer r-to-z tests without the outlier participant and found the same pattern of results (see Supplementary Material).

**Figure 3:**
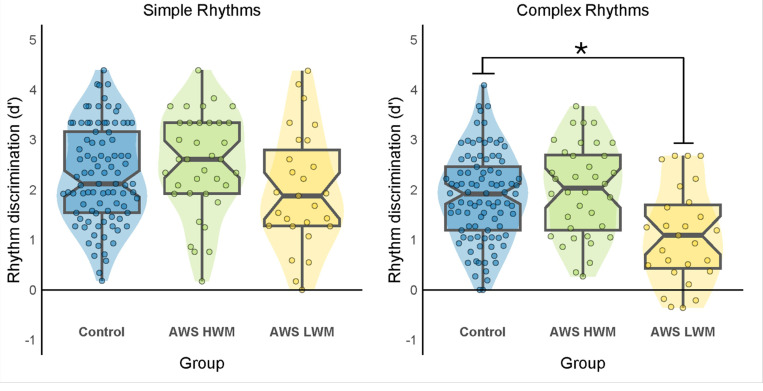
Median split on OSPAN scores to separately compare rhythm discrimination performance for AWS in high and low working memory groups to controls. Violin plots comparing rhythm discrimination performance for AWS in high and low working memory groups to controls for simple rhythms (left) and complex rhythms (right). AWS in the low working memory group showed poorer rhythm discrimination than controls for both simple rhythms and complex rhythms, however the difference was only significant for complex rhythms, indicated by the asterisk. HWM = high working memory. LWM = low working memory.

Finally, we performed a median split on OSPAN scores to create two subgroups of AWS: high working memory and low working memory. We then separately compared rhythm discrimination performance in each of the AWS subgroups to controls. The results are summarized in Fig. 3. There were no significant differences between AWS in the high working memory group and controls for either simple rhythms, t(127) = −1.132, p = 0.260, Cohen’s d = −0.222, or complex rhythms, t(127) = −0.765, p = 0.446, Cohen’s d = −0.150. Indeed, AWS in the high working memory groups performed slightly better than controls. However, AWS in the low working memory group showed poorer rhythm discrimination than controls for both simple rhythms and complex rhythms, but this decrease in performance was only significant for the complex rhythms, t(118) = 3.81, p < 0.001, Cohen’s d = 0.833 and not for the simple rhythms, t(118) = 1.361, p = 0.176, Cohen’s d = 0.298.

## Discussion

The present study compared working memory, auditory rhythm discrimination, and the association between working memory capacity and rhythm discrimination in AWS and non-stuttering adults using, as far as we are aware, the largest sample of AWS to date (AWS, n = 65; Controls, n = 94). The central hypothesis guiding this project was that AWS may be able to at least partially overcome inherent rhythm and timing limitations associated with a compromised BGTC network by leveraging an interval-based timing mechanism that does not require the generation of an internal beat, but does place greater demands on working memory. If this is the case, then AWS would be expected to show a greater positive correlation between working memory and rhythm discrimination than non-stuttering adults. To investigate this possibility, we combined data across three studies where AWS and age-matched control participants completed the same complex operation span task and the same auditory rhythm discrimination task that contrasted simple rhythms with a consistently marked beat and complex rhythms with an inconsistently marked beat.

There were four main findings. First, there was no difference in OSPAN scores between AWS and control participants across all three studies. Operation span is a complex verbal span task that shares commonalities with other span tasks: the overall premise is that the to-be-recalled series of letters are separated by a distracting task, in this case, simple math problems. Thus, the operation span task involves a phonological working memory component, namely the letters to be recalled. The current results showed no significant difference between the two groups, and moreover, a similar range of OSPAN scores for both groups. Additionally, the math accuracy was near ceiling and did not differ between groups. Data from other studies of working memory in AWS paints a mixed picture. This is noteworthy because previous studies have found lower working memory scores in AWS compared to controls using the same OSPAN task and partial score measure^74,75^.

Working memory has been of general interest in understanding the neurobiological basis of stuttering. Theoretical models of speech production hypothesize that working memory deficits may contribute to the development of stuttering^76–85^, potentially even representing a neural subtype of stuttering related to *phonological* working memory in particular^86,87^. Specifically, The Gradient Order Directions into Velocities of Articulators (GODIVA) model proposes that stuttering arises from a malfunction in the cortico-basal ganglia-thalamo-cortical circuit involved in speech production^86,88,89^. This circuit comprises two loops: a motor loop that initiates speech motor programs at the proper times, and a planning loop involving phonological working memory that buffers upcoming phonological content and translates it into speech motor programs. The planning (or phonological) loop^90–92^ temporarily stores the upcoming phonemes before they are produced overtly, similar to the phonological encoding stage proposed by Levelt^93^. This phonological loop supports sub-vocal rehearsal, a means of maintaining the information in phonological working memory^94–96^. Research into the role of the phonological loop supports the idea that stuttering may not be isolated to a speech initiation and/or articulation deficit but may also involve deficits at a pre-articulatory level (for reviews, see^76,97,98^). Phonological working memory operates between the stages of language formulation and motor control, and for fluent speech production, functions to temporarily store upcoming syllables for eventual production^86^. Tasks like nonword repetition are often used to measure phonological working memory, which requires the assembly and correct ordering of syllables prior to speech production. Such tasks are predictive of a wide array of cognitive constructs, including verbal working memory, and associated disorders^71,74,99,100^. Studies of nonword repetition and other tasks that engage phonological working memory have found poorer performance by AWS^85,101–105^ compared to nonstuttering adults.

We did not find any OSPAN differences between AWS and controls in any of the three studies. This is partially consistent with the albeit limited previous reports of OSPAN scores in AWS. Eichorn et al.)^74^ found no differences in operation span between AWS and controls, though AWS exhibited subtle deficits on a spatial span task. Tichenor et al.^75^ found the opposite: AWS exhibited lower operation span relative to controls, but no differences in spatial span. It is difficult to compare our results with these previous studies because they either included a small sample size^74^ or modified versions of the span tasks^75^. Additionally, both studies used somewhat different diagnostic and inclusion criteria from each other, and from the current study. Stuttering is a highly heterogeneous disorder^106–112^, and thus it is not surprising that group differences may vary from one group of AWS to another.

Second, auditory rhythm discrimination performance showed a robust beat-based advantage, with simple rhythms with a consistently marked beat better discriminated than complex rhythms with an inconsistently marked beat - a finding that has been consistently observed across numerous studies^18,19,24,26,33,48,49^. Of note, the expected beat-based advantage^18,72^ was found for both AWS and controls, whereby it was more challenging to detect changes in complex rhythms that lack a distinct periodic beat compared to simple rhythms with a consistent beat.

Third, overall AWS showed slightly worse rhythm discrimination compared for complex rhythms, but not simple rhythms. Although no other groups to our knowledge have used the same rhythm discrimination paradigm to investigate developmental stuttering, we have now observed the same pattern in four studies (one with children^26^ and the three combined here) for a paradigm that has been widely validated in a number of other studies involving different populations. The current investigation thus offers some perspective on the inconsistent findings across rhythm and timing performance by stutterers more generally. Although the observed group rhythm difference is small, the fact that this trend is found for studies conducted at different sites, using different equipment (during fMRI scanning vs. on a computer), different task lengths, and different participants, but with the same overall design and subsets of the rhythmic stimuli, suggests that the AWS complex rhythm deficit is a genuine effect. Furthermore, considering the heterogeneous nature of stuttering, it is not surprising that group comparisons alone may not provide a full picture of potential deficits.

Finally, as a step towards better understanding individual differences, AWS showed a significantly greater positive correlation between working memory and rhythm discrimination compared to the control group, which was true for both simple and complex rhythms. Moreover, when comparing rhythm discrimination performance between controls and AWS with high vs. low working memory scores, there were no differences between AWS with high working memory scores and controls. However, AWS with low working memory scores showed significantly poorer performance compared to controls, especially with complex rhythms. These results are consistent with the central hypothesis that some AWS compensate for a disruption in the BGTC network and beat-based timing by leveraging the cerebellum and a working-memory dependent interval-timing mechanism to make discrimination judgments.

Thus, with respect to advancing understanding of developmental stuttering, AWS in particular may leverage a distinct working-memory-dependent interval-based timing mechanism to detect rhythmic deviations, rather than having to rely on a compromised beat-based timing circuit. Their resulting success, however, may be limited by their working memory capacity. Such an account may help to explain the lack of consistent or robust rhythm differences between individuals who do and do not stutter, despite observed structural and functional abnormalities in brain regions associated with rhythm and timing. Because of the inherent differential reliance on working memory for beat-based vs. internal-based timing, these findings suggest a potential behavioral signature that may account for some of the individual differences in rhythmic ability of people who stutter.

Beat-based timing involves perceiving and synchronizing with a regular, underlying pulse or beat within a rhythmic pattern. Individuals using this strategy focus on the regular beats that occur at consistent intervals and may naturally gravitate towards beat-based timing when exposed to regular metrical rhythms. Interval-based timing involves perceiving and producing rhythms by focusing on the durations of intervals between events (such as notes or beats) rather than an underlying regular pulse. Individuals track the absolute duration of time intervals between successive events, and the emphasis is on monitoring the sequence and length of intervals rather than fitting them to an ongoing beat. Different neural mechanisms are involved in interval-based timing compared to beat-based timing, with greater activation in regions like the prefrontal cortex and cerebellum, which are associated with precise timing and cognitive control. The cerebellum is increasingly recognized as a key area involved in working memory through its connection to frontal cortical regions. Working memory is required to maintain interval representations when performing timing tasks, especially for longer durations or complex sequences. Developmentally, improvements in timing task performance align with working memory maturation: children with better working memory tend to have more consistent time estimates for example^113,114^. In neurodevelopmental disorders such as Attention-Deficit/Hyperactivity Disorder (ADHD), Autism Spectrum Disorder (ASD), and stuttering, dysfuntion in the timing circuit is often reported^115,116^. Cerebellar compensation for impaired basal ganglia timing has been proposed for stuttering^5^, while in ASD and ADHD increased timing variability has been linked to cognitive and cerebellar differences^113,117^. Importantly, working memory capacity interacts with these processes, highlighting that cognitive context shapes how timing is learned and performed^113,114^.

We note however that though the two parallel timing systems of the basal ganglia-centric beat-based rhythm timing vs. cerebellar absolute timing for isolated intervals have been proposed^48,49^. Neuroimaging studies have also shown that the boundary between systems is not absolute: cerebellum and BG often co-activate in timing tasks^114^ and dual-task studies suggest interaction between them^118^. For example, even beat-based tasks engage cerebellar networks when demands on prediction or attention increase. Relevant to this point, the control group also showed greater association between rhythm discrimination performance and working memory when the rhythms were more complex. Conversely, the cerebellum can participate in supra-second timing via the cerebro-cerebellar loop^118^. Open questions remain about how these circuits coordinate and how they differentially contribute to implicit (prediction-based) vs. explicit timing tasks in people who stutter relative to those who do not. In future studies, the relative contribution of working memory to performing beat-based timing tasks in children and adults who stutter may be examined in conjunction with measures such as stuttering severity and cerebellar connectivity. Delving further into the function of the BG and cerebellar timing circuits and their relative contributions to beat-based and interval-based timing tasks in the context of stuttering severity or other behavioral measures (e.g., executive control, attention) has the potential to help elucidate the role of timing in stuttering and related neurodevelopmental conditions.

## Conclusions

The present investigation is, as far as we are aware, the largest study comparing working memory, auditory rhythm discrimination, and the association between working memory and rhythm discrimination in adults who do and do not stutter. This work contributes to the understanding of stuttering in four ways. First, we have provided strong evidence that AWS, as a group, do not differ from their nonstuttering peers in working memory capacity, as measured by the complex operation span task. Second, we have demonstrated the expected beat-based advantage in rhythm discrimination. Specifically, simple rhythms with a consistently marked beat are better discriminated than complex rhythms with an inconsistently marked beat for both AWS and controls. Third, relative to controls, AWS consistently show similar simple rhythm discrimination performance, but slightly worse complex rhythm discrimination performance. This finding highlights both the heterogeneous nature of developmental stuttering and the potential for AWS to use an alternative interval-based timing mechanism to overcome limitations in beat-based timing associated with a compromised BGTC network.

Lastly, based on our central hypothesis, we predicted that if AWS rely more on interval-by-interval duration comparisons to discriminate rhythms than beat-based timing there would be a strong relationship between working memory and rhythm discrimination in AWS compared to adults who do not stutter. This prediction was supported by the data, which showed stronger correlations between rhythm discrimination performance and working memory scores in AWS than in controls for both simple and complex rhythms. Moreover, separate group comparison of rhythm discrimination performance for AWS with high and low working memory scores reveals no difference between controls for AWS with high working memory scores, but much poorer performance for AWS with low working memory scores compared to controls. These results support the view that AWS may mask difficulties in rhythm perception and an underlying impairment in beat-based timing by leveraging a distinct working memory dependent interval timing mechanism to discriminate rhythms, if they have the working memory capacity to do so. More broadly, interval-based timing may also be used when non-stuttering individuals are exposed to complex, non-metrical rhythms or irregular patterns, as evidenced by the positive correlation for complex rhythms when data from all three studies were combined for controls. In other words, even nonstuttering adults may increasingly rely on interval-by-interval duration strategies for more complex rhythms, but this shift is significantly more prominent in stuttering adults.

## Supplementary Material

Supplement 1

## Figures and Tables

**Table 1:** Participant demographics for individual and combined samples.

Study 1					
	Controls		AWS		
	n = 17 (6F)		n = 17 (5F)		
	Mean (SD)	Range	Mean (SD)	Range	p
Age (years)	25.14 *(7.11)*	19–44	28.19 *(10.90)*	18–53	0.342
Education (years)	15.41 *(1.84)*	13–19	16.06 *(3.01)*	12–26	0.455
OSPAN score	42.88 *(17.26)*	10–75	36.53 *(16.14)*	6–62	0.276
Musical training^a^	4.59 *(3.71)*	0–10	3.88 *(4.61)*	0–12	0.626
Study 2					
	Controls		AWS		
	n = 26 (18F)		n = 27 (3F)		
	Mean (SD)	Range	Mean (SD)	Range	p
Age (years)	25.42 *(9.73)*	18–56	25.50 *(7.30)*	19–49	0.974
Education (years)	15.52 *(2.77)*	12–24	16.30 *(2.46)*	12–24	0.285
OSPAN score	57.77 *(13.35)*	27–75	55.85 *(12.07)*	25–75	0.585
Musical training^b^	22.69 *(11.15)*	4–41	18.19 *(13.10)*	2–43	0.184
Study 3					
	Controls		AWS		
	n = 51 (18F)		n = 21 (8F)		
	Mean (SD)	Range	Mean (SD)	Range	p
Age (years)	29.08 *(9.97)*	18–60	31.62 *(10.30)*	18–55	0.333
Education (years)	16.86 *(2.80)*	12–23	17.31 *(2.42)*	12–22	0.525
OSPAN score	58.20 *(14.19)*	9–75	57.95 *(10.69)*	32–73	0.944
Musical training^b^	16.91 (9.46*)*	2–36	19.74 *(11.01)*	2–43	0.276
Combined					
	Controls		AWS		
	n = 94 (42F)		n = 65 (16F)		
Study 1	Controls		AWS		
	n = 17 (6F)		n = 17 (5F)		
	Mean (SD)	Range	Mean (SD)	Range	p
Age (years)	27.36 *(9.54)*	18–60	28.18 *(9.56)*	18–55	0.593
Education (years)	16.23 *(2.71)*	12–24	16.56 *(2.62)*	12–26	0.441
OSPAN score	55.31 *(15.55)*	9–75	51.48 *(15.53)*	6–75	0.128
Musical training^c^	18.86 *(10.36)*	2–41	18.86 *(12.13)*	2–43	1.00

a Self-reported years of formal music training.

b Musical training factor score from Goldsmiths Musical Sophistication Index (GMSI)^70^.

c Values for mean and range calculated from studies 2 and 3 only due to differences in measurement of musical training.

## Data Availability

Data from this study may be made available for all reasonable requests to the corresponding author.

## References

[1] Zablotsky B. Prevalence and Trends of Developmental Disabilities among Children in the United States: 2009–2017. Pediatrics 144, e20190811 (2019). 31558576 10.1542/peds.2019-0811 PMC7076808

[2] Yairi E. & Ambrose N. Epidemiology of stuttering: 21st century advances. J. Fluen. Disord. 38, 66–87 (2013). 10.1016/j.jfludis.2012.11.002 PMC3687212 23773662

[3] Neef N. E. & Chang S. E. Knowns and unknowns about the neurobiology of stuttering. PLoS Biol. 22, e3002492 (2024). 38386639 10.1371/journal.pbio.3002492 PMC10883586

[4] Chang S. E. & Guenther F. H. Involvement of the Cortico-Basal Ganglia-Thalamocortical Loop in Developmental Stuttering. Frontiers Psychology 10, (2020). 10.3389/fpsyg.2019.03088 PMC6997432 32047456

[5] Chang S. E., Garnett E. O., Etchell A. & Chow H. M. Functional and Neuroanatomical Bases of Developmental Stuttering: Current Insights. Neuroscientist 25, 566–582 (2019). 30264661 10.1177/1073858418803594 PMC6486457

[6] Chang S. E., Erickson K. I., Ambrose N. G., Hasegawa-Johnson M. A. & Ludlow C. L. Brain anatomy differences in childhood stuttering. NeuroImage 39, 1333–1344 (2008). 18023366 10.1016/j.neuroimage.2007.09.067 PMC2731627

[7] Watkins K. E., Smith S. M., Davis S. & Howell P. Structural and functional abnormalities of the motor system in developmental stuttering. Brain 131, 50–59 (2007). 17928317 10.1093/brain/awm241 PMC2492392

[8] Lu C. Altered effective connectivity and anomalous anatomy in the basal ganglia- thalamocortical circuit of stuttering speakers. Cortex 46, 49–67 (2010). 19375076 10.1016/j.cortex.2009.02.017

[9] Chang S. E., Horwitz B., Ostuni J., Reynolds R. & Ludlow C. L. Evidence of Left Inferior Frontal-Premotor Structural and Functional Connectivity Deficits in Adults Who Stutter. Cereb. Cortex. 21, 2507–2518 (2011). 21471556 10.1093/cercor/bhr028 PMC3183422

[10] Chang S. E. & Zhu D. C. Neural network connectivity differences in children who stutter. Brain 136, 3709–3726 (2013). 24131593 10.1093/brain/awt275 PMC3859219

[11] Giraud A. Severity of dysfluency correlates with basal ganglia activity in persistent developmental stuttering. Brain Lang. 104, 190–199 (2008). 17531310 10.1016/j.bandl.2007.04.005

[12] Beal D. S., Gracco V. L., Brettschneider J., Kroll R. M. & De Nil L. F. A voxel-based morphometry (VBM) analysis of regional grey and white matter volume abnormalities within the speech production network of children who stutter. Cortex 49, 2151–2161 (2013). 23140891 10.1016/j.cortex.2012.08.013 PMC3617061

[13] Garnett E. O. Anomalous morphology in left hemisphere motor and premotor cortex of children who stutter. Brain 141, 2670–2684 (2018). 30084910 10.1093/brain/awy199 PMC6113637

[14] Chow H. M., Garnett E. O., Ratner N. B. & Chang S. E. Brain activity during the preparation and production of spontaneous speech in children with persistent stuttering. NeuroImage: Clin. 38, 103413 (2023). 37099876 10.1016/j.nicl.2023.103413 PMC10149502

[15] Chow H. M., Garnett E. O., Koenraads S. P. C. & Chang S. E. Brain developmental trajectories associated with childhood stuttering persistence and recovery. Dev. Cogn. Neurosci. 60, 101224 (2023). 36863188 10.1016/j.dcn.2023.101224 PMC9986501

[16] Miller H. E. A comparison of structural morphometry in children and adults with persistent developmental stuttering. Brain Commun. 5, fcad301 (2023). 38025273 10.1093/braincomms/fcad301 PMC10653153

[17] Chow H. M. & Chang S. E. White matter developmental trajectories associated with persistence and recovery of childhood stuttering. Hum. Brain Mapp. 38, 3345–3359 (2017). 28390149 10.1002/hbm.23590 PMC5632574

[18] Grahn J. A. & Brett M. Rhythm and beat perception in motor areas of the brain. J. Cogn. Neurosci. 19, 893–906 (2007). 17488212 10.1162/jocn.2007.19.5.893

[19] Grahn J. A. & McAuley J. D. Neural bases of individual differences in beat perception. NeuroImage 47, 1894–1903 (2009). 19376241 10.1016/j.neuroimage.2009.04.039

[20] Grahn J. A. & Rowe J. B. Feeling the Beat: Premotor and Striatal Interactions in Musicians and Nonmusicians during Beat Perception. J. Neurosci. 29, 7540–7548 (2009). 19515922 10.1523/JNEUROSCI.2018-08.2009 PMC2702750

[21] Kotz S. A. & Schwartze M. Cortical speech processing unplugged: a timely subcortico-cortical framework. Trends Cogn. Sci. 14, 392–399 (2010). 20655802 10.1016/j.tics.2010.06.005

[22] Merchant H., Harrington D. L. & Meck W. H. Neural Basis of the Perception and Estimation of Time. Annu. Rev Neurosci. 36, 313–336 (2013). 23725000 10.1146/annurev-neuro-062012-170349

[23] Geiser E., Notter M. & Gabrieli J. D. E. A Corticostriatal Neural System Enhances Auditory Perception through Temporal Context Processing. J. Neurosci. 32, 6177–6182 (2012). 22553024 10.1523/JNEUROSCI.5153-11.2012 PMC6622138

[24] Grahn J. A. & Brett M. Impairment of beat-based rhythm discrimination in Parkinson’s disease. Cortex 45, 54–61 (2009). 19027895 10.1016/j.cortex.2008.01.005

[25] Alm P. A. Stuttering and the basal ganglia circuits: a critical review of possible relations. J. Commun. Disord. 37, 325–369 (2004). 15159193 10.1016/j.jcomdis.2004.03.001

[26] Wieland E. A., McAuley J. D., Dilley L. C. & Chang S. E. Evidence for a rhythm perception deficit in children who stutter. Brain Lang. 144, 26–34 (2015). 25880903 10.1016/j.bandl.2015.03.008 PMC5382013

[27] Garnett E. O. Auditory rhythm discrimination in adults who stutter: An fMRI study. Brain Lang. 236, 105219 (2023). 36577315 10.1016/j.bandl.2022.105219 PMC12955694

[28] Wingate M. E. Foundations of Stuttering (Academic, 2002).

[29] Wohl M. T. The Electronic Metronome-An Evaluative Study. Br. J. Disorders Communication. 3, 89–98 (1968). 10.3109/13682826809011445 5665927

[30] Adams M. R. & Ramig P. Vocal Characteristics of Normal Speakers and Stutterers during Choral Reading. J. Speech Lang. Hear. Res. 23, 457–469 (1980). 10.1044/jshr.2302.457 7442204

[31] Ingham R. J. & Carroll P. J. Listener judgement of differences in stutterers’ nonstuttered speech during chorus- and nonchorus-reading conditions. J. Speech Hear. Res. 20, 293–302 (1977). 895099 10.1044/jshr.2002.293

[32] Glover H., Kalinowski J., Rastatter M. & Stuart A. Effect of Instruction to Sing on Stuttering Frequency at Normal and Fast Rates. Percept Mot Skills. 83, 511–522 (1996). 8902026 10.2466/pms.1996.83.2.511

[33] Chang S. E., Chow H. M., Wieland E. A. & McAuley J. D. Relation between functional connectivity and rhythm discrimination in children who do and do not stutter. NeuroImage: Clin. 12, 442–450 (2016). 27622141 10.1016/j.nicl.2016.08.021 PMC5008055

[34] Falk S., Müller T. & Bella S. D. Sensorimotor Synchronization in Stuttering Children and Adolescents. Procedia - Social Behav. Sci. 126, 206–207 (2014).

[35] Howell P., Au-Yeung J. & Rustin L. Clock and motor variances in lip-tracking: A comparison between children who stutter and those who do not. in Speech production: Motor control, brain research and fluency disorders 573–578 (Elsevier, Amsterdam, the Netherlands, (1997).

[36] Olander L., Smith A. & Zelaznik H. Evidence That a Motor Timing Deficit Is a Factor in the Development of Stuttering. J. Speech Lang. Hear Res. 53, 876–886 (2010). 20220024 10.1044/1092-4388(2009/09-0007) PMC3918902

[37] Cooper M. H. & Allen G. D. Timing Control Accuracy in Normal Speakers and Stutterers. J. Speech Hear Res. 20, 55–71 (1977). 846204 10.1044/jshr.2001.55

[38] Zelaznik H. N., Smith A., Franz E. A. & Ho M. Differences in bimanual coordination associated with stuttering. Acta. Psychol. 96, 229–243 (1997). 10.1016/s0001-6918(97)00014-0 9434590

[39] Brown C. J., Zimmermann G. N., Linville R. N. & Hegmann J. P. Variations in Self-Paced Behaviors in Stutterers and Nonstutterers. J. Speech Lang. Hear Res. 33, 317–323 (1990). 10.1044/jshr.3302.317 2359272

[40] Max L. & Yudman E. M. Accuracy and Variability of Isochronous Rhythmic Timing Across Motor Systems in Stuttering Versus Nonstuttering Individuals. J. Speech Lang. Hear Res. 46, 146–163 (2003). 12647895 10.1044/1092-4388(2003/012)

[41] Neef N. E. Right-shift for non-speech motor processing in adults who stutter. Cortex 47, 945–954 (2011). 20822768 10.1016/j.cortex.2010.06.007

[42] Zelaznik H. N., Smith A. & Franz E. A. Motor Performance of Stutterers and Nonstutterers on Timing and Force Control Tasks. J. Mot. Behav. 26, 340–347 (1994). 12719191 10.1080/00222895.1994.9941690

[43] Assaneo M. F., Ripolles P., Tichenor S. E., Yaruss J. S. & Jackson E. S. The Relationship Between Auditory-Motor Integration, Interoceptive Awareness, and Self-Reported Stuttering Severity. Frontiers Integr Neuroscience 16, (2022). 10.3389/fnint.2022.869571 PMC9120354 35600224

[44] Ivry R. Cerebellar involvement in the explicit representation of temporal information. Ann. N Y Acad. Sci. 682, 214–230 (1993). 8323114 10.1111/j.1749-6632.1993.tb22970.x

[45] Spencer R. M. C., Zelaznik H. N., Diedrichsen J. & Ivry R. B. Disrupted timing of discontinuous but not continuous movements by cerebellar lesions. Science 300, 1437–1439 (2003). 12775842 10.1126/science.1083661

[46] Ivry R. B. & Keele S. W. Timing Functions of The Cerebellum. J. Cogn. Neurosci. 1, 136–152 (1989). 23968462 10.1162/jocn.1989.1.2.136

[47] Ivry R. B. & Spencer R. M. The neural representation of time. Curr Opin. Neurobiol. 14, 225–232 (2004). 15082329 10.1016/j.conb.2004.03.013

[48] Grube M., Cooper F. E., Chinnery P. F. & Griffiths T. D. Dissociation of duration-based and beat-based auditory timing in cerebellar degeneration. Proceedings of the National Academy of Sciences 107, 11597–11601 (2010). 10.1073/pnas.0910473107 PMC2895141 20534501

[49] Teki S., Grube M., Kumar S. & Griffiths T. D. Distinct neural substrates of duration-based and beat-based auditory timing. J. Neurosci. 31, 3805–3812 (2011). 21389235 10.1523/JNEUROSCI.5561-10.2011 PMC3074096

[50] Breska A. & Ivry R. B. Double dissociation of single-interval and rhythmic temporal prediction in cerebellar degeneration and Parkinson’s disease. Proc. Natl. Acad. Sci. US A. 115, 12283–12288 (2018). 10.1073/pnas.1810596115 PMC6275527 30425170

[51] Chang S. E., Kenney M. K., Loucks T. M. J. & Ludlow C. L. Brain activation abnormalities during speech and non-speech in stuttering speakers. NeuroImage 46, 201–212 (2009). 19401143 10.1016/j.neuroimage.2009.01.066 PMC2693291

[52] Fox P. T. Brain correlates of stuttering and syllable production. A PET performance-correlation analysis. Brain 123 (Pt 10), 1985–2004 (2000). 11004117 10.1093/brain/123.10.1985

[53] Frankford S. A. The Neural Circuitry Underlying the ‘Rhythm Effect’ in Stuttering. J. Speech Lang. Hear. Res. 1–22. 10.1044/2021_JSLHR-20-00328 (2021). 33887150 PMC8740675

[54] Ingham R. J., Grafton S. T., Bothe A. K. & Ingham J. C. Brain activity in adults who stutter: Similarities across speaking tasks and correlations with stuttering frequency and speaking rate. Brain Lang. 122, 11–24 (2012). 22564749 10.1016/j.bandl.2012.04.002 PMC3372660

[55] Neumann K. The nature and treatment of stuttering as revealed by fMRI A within- and between-group comparison. J. Fluen. Disord. 28, 381–409 (2003). quiz 409–410. 10.1016/j.jfludis.2003.07.003 14643071

[56] Toyomura A., Fujii T. & Kuriki S. Effect of external auditory pacing on the neural activity of stuttering speakers. NeuroImage 57, 1507–1516 (2011). 21624474 10.1016/j.neuroimage.2011.05.039

[57] Toyomura A., Fujii T. & Kuriki S. Effect of an 8-week practice of externally triggered speech on basal ganglia activity of stuttering and fluent speakers. NeuroImage 109, 458–468 (2015). 25595501 10.1016/j.neuroimage.2015.01.024

[58] Kell C. A., Neumann K., Behrens M., von Gudenberg A. W. & Giraud A. L. Speaking-related changes in cortical functional connectivity associated with assisted and spontaneous recovery from developmental stuttering. J. Fluen. Disord. 55, 135–144 (2018). 10.1016/j.jfludis.2017.02.001 28216127

[59] De Nil L. F., Kroll R. M. & Houle S. Functional neuroimaging of cerebellar activation during single word reading and verb generation in stuttering and nonstuttering adults. Neurosci. Lett. 302, 77–80 (2001). 11290391 10.1016/s0304-3940(01)01671-8

[60] Lu M. K., Arai N., Tsai C. H. & Ziemann U. Movement related cortical potentials of cued versus self-initiated movements: double dissociated modulation by dorsal premotor cortex versus supplementary motor area rTMS. Hum. Brain Mapp. 33, 824–839 (2012). 21425396 10.1002/hbm.21248 PMC6870267

[61] Strick P. L., Dum R. P. & Fiez J. A. Cerebellum and nonmotor function. Annu. Rev. Neurosci. 32, 413–434 (2009). 19555291 10.1146/annurev.neuro.31.060407.125606

[62] Schmahmann J. D., Guell X., Stoodley C. J. & Halko M. A. The Theory and Neuroscience of Cerebellar Cognition. Annu. Rev Neurosci. 42, 337–364 (2019). 30939101 10.1146/annurev-neuro-070918-050258

[63] Koziol L. F. Consensus Paper: The Cerebellum’s Role in Movement and Cognition. Cerebellum 13, 151–177 (2014). 23996631 10.1007/s12311-013-0511-x PMC4089997

[64] Gibbon J., Church R. M. & Meck W. H. Scalar timing in memory. Ann. N Y Acad. Sci. 423, 52–77 (1984). 6588812 10.1111/j.1749-6632.1984.tb23417.x

[65] Ivry R. B. & Hazeltine R. E. Perception and production of temporal intervals across a range of durations: Evidence for a common timing mechanism. J. Exp. Psychol. Hum. Percept. Perform. 21, 3–18 (1995). 7707031 10.1037//0096-1523.21.1.3

[66] Keele S. W., Nicoletti R., Ivry R. I. & Pokorny R. A. Mechanisms of perceptual timing: Beat-based or interval-based judgements? Psychol. Res. 50, 251–256 (1989).

[67] McAuley J. D. & Jones M. R. Modeling Effects of Rhythmic Context on Perceived Duration: A Comparison of Interval and Entrainment Approaches to Short-Interval Timing. J. Exp. Psychol. Hum. Percept Perform. 29, 1102–1125 (2003). 14640833 10.1037/0096-1523.29.6.1102

[68] McAuley J. D. Perception of Time as Phase: Toward an Adaptive-Oscillator Model of Rhythmic Pattern Processing (Indiana University, 1995).

[69] Riley G. in SSI-4: Stuttering Severity Instrument - Fourth Edition. (eds Austin) (PRO-ED, 2009).

[70] Mullensiefen D., Gingras B., Stewart L. & Ji J. Goldsmiths Musical Sophistication Index (Gold-MSI) v1.0: Technical Report and Documentation Revision 0.3.

[71] Unsworth N., Heitz R. P., Schrock J. C. & Engle R. W. An automated version of the operation span task. Behav Res. Methods. 37, 498–505 (2005). 16405146 10.3758/bf03192720

[72] Povel D. J. & Essens P. Perception of Temporal Patterns. Music Percept 2, 411–440 (1985). 10.3758/bf03207132 3991313

[73] Macmillan N. A. & Creelman C. D. Detection Theory: A User’s Guide, 2nd Ed. xix, 492 Lawrence Erlbaum Associates Publishers, Mahwah, NJ, US, (2005).

[74] Eichorn N., Hall J. & Marton K. Complex working memory in adults with and without stuttering disorders: Performance patterns and predictive relationships. J. Fluen. Disord. 77, 105993 (2023). 10.1016/j.jfludis.2023.105993 37406551

[75] Tichenor S. E., Hampton Wray A., Ravizza S. M. & Yaruss J. S. Individual differences in attentional control predict working memory capacity in adults who stutter. J. Commun. Disord. 100, 106273 (2022). 36274445 10.1016/j.jcomdis.2022.106273

[76] Bajaj A. Working memory involvement in stuttering: Exploring the evidence and research implications. J. Fluen. Disord. 32, 218–238 (2007). 10.1016/j.jfludis.2007.03.002 17825670

[77] Anderson J. D. & Wagovich S. A. Relationships among linguistic processing speed, phonological working memory, and attention in children who stutter. J. Fluen. Disord. 35, 216–234 (2010). 10.1016/j.jfludis.2010.04.003 PMC2939037 20831969

[78] Anderson J. D., Wagovich S. A. & Brown B. T. Phonological and Semantic Contributions to Verbal Short-Term Memory in Young Children With Developmental Stuttering. J. Speech Lang. Hear Res. 62, 644–667 (2019). 30950742 10.1044/2018_JSLHR-S-18-0039 PMC6802901

[79] Anderson J. D., Wagovich S. A. & Hall N. E. Nonword repetition skills in young children who do and do not stutter. J. Fluen. Disord. 31, 177–199 (2006). 10.1016/j.jfludis.2006.05.001 PMC2443718 16814376

[80] Hakim H. B. & Ratner N. B. Nonword repetition abilities of children who stutter: an exploratory study. J. Fluen. Disord. 29, 179–199 (2004). 10.1016/j.jfludis.2004.06.001 15458830

[81] Pelczarski K. M. & Yaruss J. S. Phonological memory in young children who stutter. J. Commun. Disord. 62, 54–66 (2016). 27280891 10.1016/j.jcomdis.2016.05.006

[82] Weber-Fox C., Spruill J. E., Spencer R. & Smith A. Atypical neural functions underlying phonological processing and silent rehearsal in children who stutter. Dev. Sci. 11, 321–337 (2008). 18333985 10.1111/j.1467-7687.2008.00678.x PMC2673811

[83] Oyoun H. A., Dessouky H., Shohdi S. & Fawzy A. Assessment of Working Memory in Normal Children and Children Who Stutter. in (2010).

[84] Sasisekaran J. & Byrd C. Nonword repetition and phoneme elision skills in school-age children who do and do not stutter: Nonword repetition and phoneme elision in CWS. Int. J. Lang. Communication Disorders. 48, 625–639 (2013). 10.1111/1460-6984.12035 PMC3992859 24165360

[85] Byrd C. T., McGill M. & Usler E. Nonword repetition and phoneme elision in adults who do and do not stutter: Vocal versus nonvocal performance differences. J. Fluen. Disord. 44, 17–31 (2015). 10.1016/j.jfludis.2015.01.004 25680736

[86] Guenther F. H. Neural Control of Speech (MIT Press, 2016).

[87] Rowe H. P. Evidence for planning and motor subtypes of stuttering based on resting state functional connectivity. Brain Lang. 253, 105417 (2024). 38703523 10.1016/j.bandl.2024.105417 PMC11147703

[88] Civier O., Bullock D., Max L. & Guenther F. H. Computational modeling of stuttering caused by impairments in a basal ganglia thalamo-cortical circuit involved in syllable selection and initiation. Brain Lang. 126, 263–278 (2013). 23872286 10.1016/j.bandl.2013.05.016 PMC3775364

[89] Bohland J. W., Bullock D. & Guenther F. H. Neural Representations and Mechanisms for the Performance of Simple Speech Sequences. J. Cogn. Neurosci. 22, 1504–1529 (2010). 19583476 10.1162/jocn.2009.21306 PMC2937837

[90] Baddeley A. Working Memory, xi, 289 (Clarendon Press/Oxford University Press, New York, NY, US, (1986).

[91] Baddeley A. Working memory: looking back and looking forward. Nat Rev Neurosci. 4, 829–839 (2003). 14523382 10.1038/nrn1201

[92] Baddeley A. Working memory: theories, models, and controversies. Annu. Rev Psychol. 63, 1–29 (2012). 21961947 10.1146/annurev-psych-120710-100422

[93] Levelt W. J. M., Roelofs A. & Meyer A. S. A theory of lexical access in speech production. BEHAVIORAL BRAIN SCIENCES 76 (1999). 10.1017/s0140525x99001776 11301520

[94] Bishop D. V. & Robson J. Unimpaired short-term memory and rhyme judgement in congenitally speechless individuals: Implications for the notion of ‘articulatory coding’. Q. J. Experimental Psychol. A: Hum. Experimental Psychol. 41, 123–140 (1989).

[95] Caplan D., Rochon E. & Waters G. S. Articulatory and phonological determinants of word length effects in span tasks. Q. J. Exp. Psychol. A. 45, 177–192 (1992). 1410554 10.1080/14640749208401323

[96] Gathercole S. & Alloway T. P. Working Memory and Learning: A Practical Guide for Teachers SAGE,. (2008).

[97] Anderson J. D. & Ofoe L. C. The Role of Executive Function in Developmental Stuttering. Semin Speech Lang. 40, 305–319 (2019). 31311055 10.1055/s-0039-1692965 PMC6910129

[98] Sasisekaran J. Exploring the Link between Stuttering and Phonology: A Review and Implications for Treatment. Semin Speech Lang. 35, 95–113 (2014). 24782273 10.1055/s-0034-1371754

[99] Avery E. W. Distributed Patterns of Functional Connectivity Predict Working Memory Performance in Novel Healthy and Memory-impaired Individuals. J. Cogn. Neurosci. 32, 241–255 (2020). 31659926 10.1162/jocn_a_01487 PMC8004893

[100] Daneman M. Individual differences in reading skills. in Handbook of reading research, Vol. 2. 512–538 (Lawrence Erlbaum Associates, Inc, Hillsdale, NJ, US, (1991).

[101] Sasisekaran J. Nonword repetition and nonword reading abilities in adults who do and do not stutter. J. Fluen. Disord. 38, 275–289 (2013). 10.1016/j.jfludis.2013.06.001 PMC3834355 24238389

[102] Sasisekaran J. & Weisberg S. Practice and retention of nonwords in adults who stutter. J. Fluen. Disord. 41, 55–71 (2014). 10.1016/j.jfludis.2014.02.004 PMC4156135 25173457

[103] Coalson G. A. & Byrd C. T. Nonword repetition in adults who stutter: The effects of stimuli stress and auditory-orthographic cues. PLOS ONE. 12, e0188111 (2017). 29186179 10.1371/journal.pone.0188111 PMC5706734

[104] Byrd C. T., Vallely M., Anderson J. D. & Sussman H. Nonword repetition and phoneme elision in adults who do and do not stutter. J. Fluen. Disord. 37, 188–201 (2012). 10.1016/j.jfludis.2012.03.003 22682320

[105] Namasivayam A. K., van Lieshout P. & De Nil L. Bite-block perturbation in people who stutter: Immediate compensatory and delayed adaptive processes. J. Commun. Disord. 41, 372–394 (2008). 18405914 10.1016/j.jcomdis.2008.02.004

[106] Yairi E. & Ambrose N. G. in Early Childhood Stuttering for Clinicians by Clinicians. (eds Austin) (PRO-ED, 2005).

[107] Brignell A. A systematic review of interventions for adults who stutter. J. Fluen. Disord. 64, 105766 (2020). 10.1016/j.jfludis.2020.105766 32438123

[108] Tichenor S. E. & Yaruss J. S. Variability of Stuttering: Behavior and Impact. Am. J. Speech Lang. Pathol. 30, 75–88 (2021). 33197323 10.1044/2020_AJSLP-20-00112 PMC8740569

[109] Tichenor S. E. & Yaruss J. S. Stuttering as Defined by Adults Who Stutter. J. Speech Lang. Hear. Res. 62, 4356–4369 (2019). 31830837 10.1044/2019_JSLHR-19-00137

[110] Tichenor S. & Yaruss J. S. A Phenomenological Analysis of the Experience of Stuttering. Am. J. Speech Lang. Pathol. 27, 1180–1194 (2018). 30347062 10.1044/2018_AJSLP-ODC11-17-0192

[111] Tichenor S. E., Herring C. & Yaruss J. S. Understanding the Speaker’s Experience of Stuttering Can Improve Stuttering Therapy. Top. Lang. Disord. 42, 57–75 (2022). 35757374 10.1097/tld.0000000000000272 PMC9231935

[112] Constantino C. D., Leslie P., Quesal R. W. & Yaruss J. S. A preliminary investigation of daily variability of stuttering in adults. J. Commun. Disord. 60, 39–50 (2016). 26945438 10.1016/j.jcomdis.2016.02.001

[113] Brenner L. A. Time reproduction performance is associated with age and working memory in high functioning youth with autism spectrum disorder. Autism Res. 8, 29–37 (2015). 25078724 10.1002/aur.1401 PMC4312276

[114] Buzi G., Eustache F., Droit-Volet S., Desaunay P. & Hinault T. Towards a neurodevelopmental cognitive perspective of temporal processing. Commun. Biol. 7, 1–18 (2024). 39143328 10.1038/s42003-024-06641-4 PMC11324894

[115] Lense M. D., Ladanyi E., Rabinowitch T. C., Trainor L. & Gordon R. Rhythm and timing as vulnerabilities in neurodevelopmental disorders. Phil Trans. R Soc. B. 376, 20200327 (2021). 34420385 10.1098/rstb.2020.0327 PMC8380970

[116] Ladányi E., Persici V., Fiveash A., Tillmann B. & Gordon R. L. Is atypical rhythm a risk factor for developmental speech and language disorders? Wiley Interdiscip Rev Cogn. Sci. 11, e1528 (2020). 32244259 10.1002/wcs.1528 PMC7415602

[117] Valera E. M. Neural substrates of impaired sensorimotor timing in adult attention-deficit/hyperactivity disorder. Biol. Psychiatry. 68, 359–367 (2010). 20619827 10.1016/j.biopsych.2010.05.012 PMC2917236

[118] Boven E. & Cerminara N. L. Cerebellar contributions across behavioural timescales: a review from the perspective of cerebro-cerebellar interactions. Front. Syst. Neurosci. 17, 1211530 (2023). 37745783 10.3389/fnsys.2023.1211530 PMC10512466

