# Supplementary material for: Differential contribution of working memory to auditory rhythm discrimination in stuttering and nonstuttering adults
Source: Res Sq. 2025 Jun 11:rs.3.rs-6686913. Preprint. [Version 1] doi: 10.21203/rs.3.rs-6686913/v1 (PMC12204478; doi:10.21203/rs.3.rs-6686913/v1)
Supplement: Supplement 1 [file NIHPPRS6686913v1-supplement-1.pdf]

Supplementary Files

This is a list of supplementary files associated with this preprint. Click to download.

- BBAWMSuppfinal.docx
